# Higher Level of Fatty Acid Synthase Enzyme Predicts Lower Rate of Completing Debulking Surgery in Epithelial Ovarian Cancer

**DOI:** 10.31557/APJCP.2020.21.10.2859

**Published:** 2020-10

**Authors:** Gatot Nyarumenteng Adhipurnawan Winarno, Yudi Mulyana Hidayat, Setiawan Soetopo, Sofie Rifayani Krisnadi, Maringan Diapari Lumban Tobing, Syahrul Rauf

**Affiliations:** 1 *Department of Obstetrics and Gynecology, Faculty of Medicine, Padjadjaran University, Bandung, Indonesia. *; 2 *Department of Radiology, Faculty of Medicine, Padjadjaran University, Bandung, Indonesia. *; 3 *Department of Obstetrics and Gynecology, Faculty of Medicine, Hasanuddin University, Makasar, Indonesia. *

**Keywords:** Fatty acid synthase enzyme, primary debulking surgery, ovarian cancer

## Abstract

**Background:**

The most dominant histopathologic type of ovarian cancer is epithelial ovarian cancer (EOC). Primary debulking surgery determines the treatment success and prognosis of advanced stage EOC. To maintain survival and progression, cancer cells need fatty acid synthase enzyme (FASN). The aim of this study was to evaluate preoperative serum FASN and CA 125 as predictors of primary debulking surgery results in patients with EOC.

**Methods:**

An observational cross-sectional study was performed on consecutive patients who underwent debulking surgery for suspected ovarian cancer at Dr. Hasan Sadikin Hospital Bandung from 2017 to 2019. Before debulking surgery, blood samples were examined for the serum levels of FASN and CA 125 using ELISA.

**Results:**

There were 53 patients enrolled in this study. Compared with the optimal debulking surgery group, the significant suboptimal debulking surgery group had significantly lower mean serum levels of FASN (0.46 ± 0.144 vs. 0.36 ± 0.128, p = 0.012) and CA 125 (964.22 ± 1722.5 vs. 264.98 ± 251.8, p = 0.002). The cutoff value was highest for the combination of FASN and CA 125 [410.06, area under the curve (AUC) = 77.5% (95% CI 65.5% to 81.9%, p = 0.001)] than for FASN alone [0.375, AUC = 71.3% (95% CI 56.8% to 85.8%, p = 0.009)] and CA 125 alone [222.5, AUC = 75.3% (95% CI 62.5% to 88.1%, p =0.002)].

**Conclusion:**

The serum levelof FASN was correlated with suboptimal debulking surgery.

## Introduction

Ovarian cancer is the most fatal malignancy in women, with a reported incidence of 239,000 each year (Coalition, 2018). In Indonesia, ovarian cancer is the second most frequent reproductive malignancy in women after cervical cancer.(Andrijono and Nurana, 2012) Epithelial ovarian cancer (EOC) is the most dominant type and has four histologic subtypes, including serous (75%–80%), mucinous (10%), and clear cell and endometrioid (10%).(Berek et al., 2014; Reid et al., 2017). In advanced stage EOC, the size of residual tumor after primary debulking surgery determines the success and prognosis of adjuvant chemotherapy (Chesnais et al., 2017). Primary debulking surgery of all visible macroscopic tumors has been the standard treatment for all eligible patients with EOC.(Gorodnova et al., 2018). Debulking surgery is deemed optimal when the remaining tumor is <1 cm or suboptimal when the remaining tumor is >1 cm (Chesnais et al., 2017). The survival rate of ovarian cancer was reported to be prolonged by 18–39 months in patients who have received optimal debulking surgery (Shashikant and Kesterson Joshua, 2009).

The commonly used tumor marker for ovarian cancer screening is CA 125 (Mousavi et al., 2010). Several studies have used preoperative serum CA 125 level to predict the results of EOC debulking surgery (Prat, 2014). In 2010, Kang et al reported that preoperative CA 125 levels above 500 IU/mL increased the occurrence of suboptimal debulking surgery (Kang et al., 2010). However, another study in 2018 reported that preoperative CA 125 levels >100U/ mL increased the likelihood of suboptimal debulking surgery, but it also showed the inability of CA 125 to accurately predict optimal debulking (Chesnais et al., 2017). Therefore, there is a need for another parameter to better predict the results of primary debulking surgery. Fatty acids play an important role in the development of cancer cells via several mechanisms, one of which is by enhancing de novo lipogenesis. The increase in fatty acid synthesis in tumor cells is indicated by a significant elevation in the expression and activation of a number of enzymes functioning in the lipogenic pathway, one of which is fatty acid synthase (FASN). FASN is the main enzyme that produces palmitate during de novo lipogenesis, which produces saturated acids and monosaturated fatty acids that support cancer cell survival during oxidative stress that is supposed to kill the cells and limit the absorption of chemotherapy drugs, thereby, creating chemotherapy drug resistance (Ameer et al., 2014) 

As a predictor of the results of debulking surgery, CA 125 has been used but has low sensitivity and specificity, whereas FASN has not been used. Moreover, the combination of both has not been analyzed as a predictor in EOC. Therefore, this study aimed to investigate the use of preoperative serum FASN and CA 125 as predictors of the results of primary debulking surgery in patients with advanced stage (II–III) EOC.

**Figure 1:**
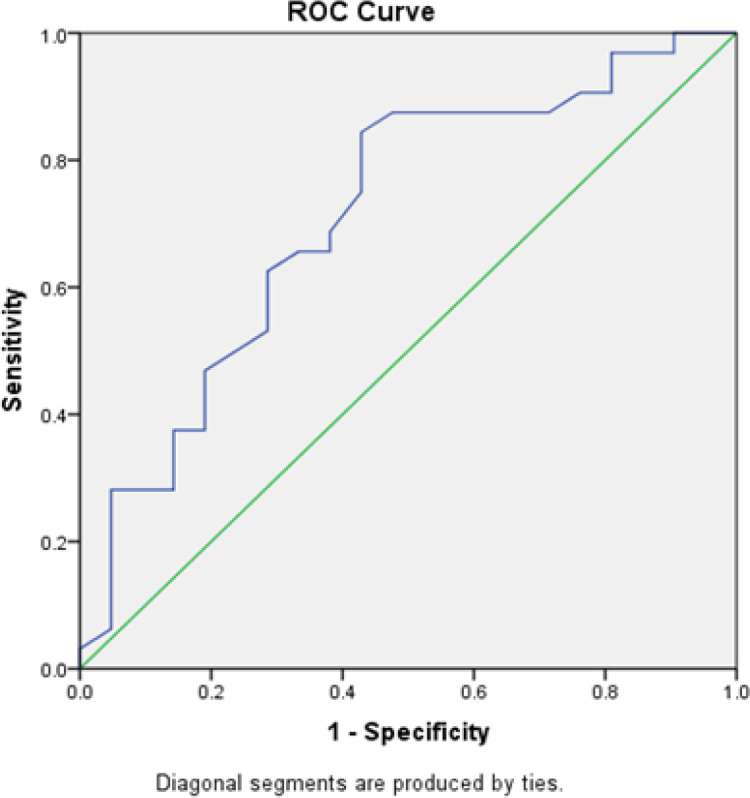
FASN with Debulking. The ROC method obtained an AUC value of 71.3% (p = 0.009) for FASN. Results showed that FASN can correctly predict debulking in 38 of 53 patients

**Table 1:** Background Characteristics of the Study Population

Variable	Groups		p value
	Suboptimal debulking N = 32	Optimal debulking N = 21	
Age (years)			0.131
Mean ± SD	50.09 ± 9.686	45.23 ± 13.374	
Median	49.5	51	
Range (min–max)	26.00–64.00	22.00–71.00	
Parity			0.979
0	7 (21.9%)	2 (9.5%)	
1	4 (12.5%)	8 (38.1%)	
2	8 (25.0%)	1 (4.8%)	
>3	13 (40.6%)	10 (47.6%)	
Height			0.164
Mean ± SD	153.09 ± 5.348	150.80 ± 4.285	
Median	152	151	
Range (min–max)	141.00–170.00	141.00–158.00	
Weight			0.056
Mean ± SD	56.25 ± 8.925	51.19 ± 9.825	
Median	56	51	
Range (min–max)	29.00–88.00	29.00–70.00	
BMI			0.175
Mean ± SD	21.44 ± 4.259	19.90 ± 3.501	
Median	21.2	20.2	
Range (min–max)	10.40–34.70	10.40–26.20	
Ascites			0.195
Mean ± SD	1892.50 ± 3126.565	2281.90 ± 5117.13	
Median	575	300	
Range (min–max)	10.00–15000.00	20.00–18000.00	
Bleeding			0.114
Mean ± SD	1312.10 ± 920.868	1357.14 ± 1826.08	
Median	1000	800	
Range (min–max)	100.00–4000.00	200.00–8000.00	
Stage			0.149
II	5 (15.6%)	10 (47.6%)	
III	20 (62.5%)	11 (52.4%)	
IV	7 (21.9%)	0 (0.0%)	
Histopathology			0.873
Serosa	13 (40.6%)	6 (28.6%)	
Mucinous	7 (21.9%)	10 (47.6%)	
Endometrioid	7 (21.9%)	2 (9.5%)	
Clear cell	5 (15.6%)	3 (14.3%)	

SD, standard deviation; Normally distributed numerical data, such as age, postoperative weight, and IMT, were analyzed using unpaired t-test, whereas nonnormally distributed data, such as height, preoperative weight, and bleeding, were analyzed using Mann–Whitney U-test. Categorical data, such as parity, stage, and histopathology, were analyzed using Kolmogorov–Smirnov test.

**Figure 2:**
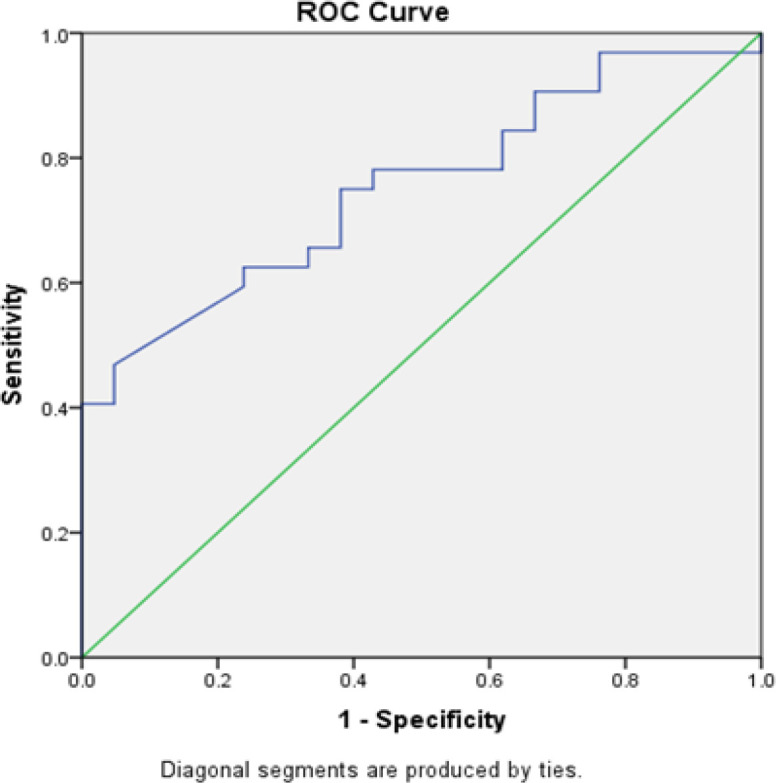
CA 125 with Debulking. The ROC method obtained an AUC value of 75.3% (p = 0.002) for CA 125. Results showed that CA 125 can correctly predict debulking in 40 of 53 patients

**Table 2:** FASN and CA 125 levels in the Suboptimal and Optimal Debulking Groups

Variable	Groups		p value
	Suboptimal debulking N = 32	Optimal debulking N = 21	
FASN (ng/mL)			0.012
Mean ± SD	0.46 ± 0.144	0.36 ± 0.128	
Median	0.44	0.34	
Range (min–max)	0.21–0.77	0.17–0.73	
CA 125 (U/mL)			0.002
Mean ± SD	964.22 ± 1722.532	264.98 ± 251.883	
Median	600	132.7	
Range (min–max)	4.29–9934.00	5.10–701.00	

The normally distributed CA 125 levels were analyzed using Mann–Whitney U-test. The normally distributed FASN levels were analyzed using unpaired t-test.

**Figure 3:**
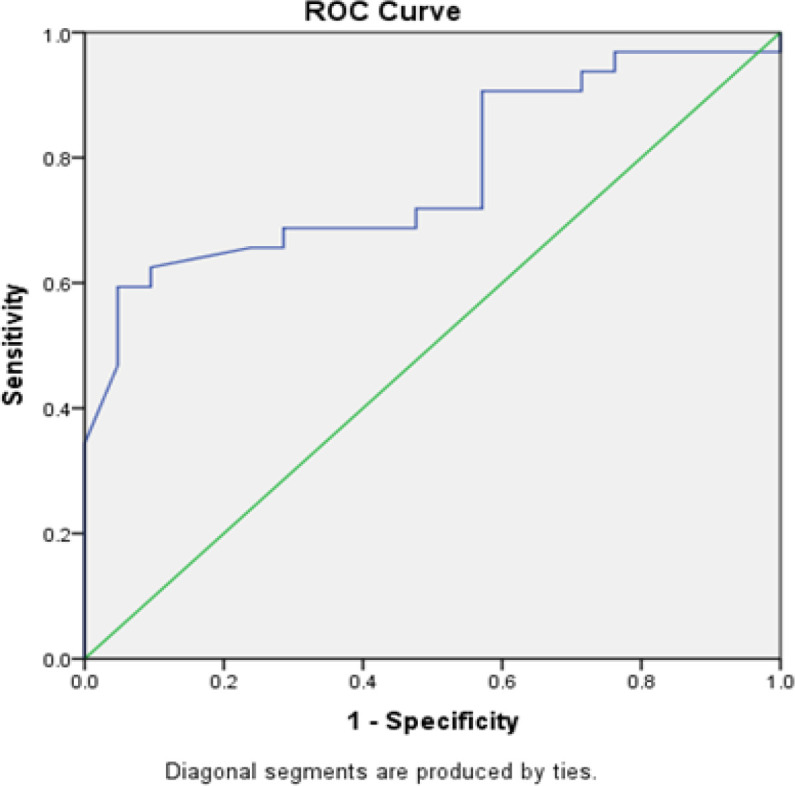
Combination of FASN and CA 125 with Debulking. The ROC method obtained an AUC value of 77.5% (p = 0.001) for the combination of FASN and CA 125. Results showed that the combination of FASN and CA 125 can correctly predict debulking in 41 of 53 patients

**Table 3:** Cutoff Points of FASN and CA 125 Levels and the Combination of Both

Variable	Debulking		P value
	Suboptimal	Optimal	
	N = 32	N = 21	
FASN			0.028
>0.375	22 (68.8%)	8 (38.1%)	
<0.375	10 (31.3%)	13 (61.9%)	
CA 125			0.007
>222.50	24 (75.0%)	8 (38.1%)	
<222.50	8 (25.0%)	13 (61.9%)	
Combination of FASN and CA 125			0.004
>471.06	22 (68.8%)	6 (28.6%)	
<471.06	10 (31.3%)	15 (71.4%)	
Range (min–max)	4.29–9934.00	5.10–701.00	

Categorical data were analyzed using chi-square test. The cutoff points were determined by ROC curves analysis

## Materials and Methods

*Study design*

An observational analysis with a cross-sectional design study was carried out at Hasan Sadikin Hospital, Bandung, Indonesia, and at the Laboratory of Molecular Genetics in Padjadjaran University from July 2017 to March 2019.

*Ethics clearance*

The research ethics committee of the Universitas Padjajaran Bandung approved this study (Ref 0518010184). All participants of this study gave written informed consent and were guaranteed that their clinical data will be handled with full confidentiality.

*Study population*

Patients who underwent debulking surgery for suspected primary EOC stage II–IV were included in this study. We collect blood sample from the patient before debulking operation. The exclusion criteria were the presence of chronic diseases or other tumors, damaged or unassessable histopathologic preparations damaged, or inoperability. There were 53 patients included in this study.

The sample size was determined based on the research objectives and the data type. In the present study, a non-spe cific design was used; the study included unpaired numerical categorical data. The sample size was determined based on 95% confidence level and 95% power. By using the formula for determining the sample size for unpaired numerical categorical analytical data, the sample size formula used was as follows:

$n_{1}=n_{2}=2{\left(\frac{\left(Z_{\alpha }+X_{\beta }\right)}{X_{1}-X_{2}}\right)}^{2}$

Z_α_ = alpha standard deviation

Z_β_= beta standard deviation

S = combined standard deviation.

X_1_−X_2_ = Difference between the minimum mean is considered significant.

which was as follows: 

$S_{g}^{2}=\frac{\left[S_{1}^{2}\times \left(n_{1}-1\right)+S_{2}^{2}(n_{2}-1)\right]}{n_{1}+n_{2}-2}$

Type 1 error was set at 5%, and the hypothesis was two-way; thus, Z_α_ = 1.96. 

Type 2 error was set at 5%, and the hypothesis was two-way; thus, Z_β_ = 1.64 

Information:

Z_α_, Z_β_ = the Z deviation value obtained from the normal/standard distribution table for the confidence level and the selected parameter

s = standard deviation

d = X_1_−X_2_, namely, the magnitude of the difference in average

The mean and standard deviation amount was determined based on the standardized range (|max−min|/SD = 1). Based on this formula, the values were entered into the sample size formula as follows:

$n_{1}=n_{2}=2{\left(\frac{(Z_{\alpha }+X_{\beta })S}{X_{1}-X_{2}}\right)}^{2}$

In the present study, there were two groups, namely, suboptimal debulking and optimal debulking; therefore, 26.03 × 2 = 52.06 samples ≈ 53. Hence, the minimal sample size required for this study was 53. 

*Measurement of FASN and CA 125 levels*

The preoperative serum levels of FASN (ng/mL) and CA 125 (U/mL) were measured by enzyme-linked immunosorbent assay kits (cloud-clone corp and Siemens Advia Centaur Xpt, respectively) at the Laboratory of Molecular Genetics in Universitas Padjajaran, according to the manufacturers’ instructions. Blood samples were left to clot for two hours at room temperature or 4°C overnight. Thereafter, the blood samples were centrifuged for 20 minutes and stored at −20°C to −80°C for subsequent use. The authors did not receive any funding resources for this study.

*Statistical analysis*

We analyzed data using SPSS ver 24.0. Univariate analysis was used to generate the frequencies and percentages of categorical variables. Numerical data were analyzed by unpaired T-test for normally distributed data or by the Mann–Whitney U-test for nonnormally distributed data. Categorical data were analyzed by the Chi-square test, with the alternatives Kolmogorov Smirnov or Fisher’s exact test, if the terms of the Chi-square were not met. Multivariate analysis using binary logistic regression was used to determine the predictors of debulking surgery. Receiver operating characteristic curve (ROC) analysis was used to obtain the cutoff and the area under the curve (AUC) values. Level of statistical significance was set at a p value of <0.05.

## Results

*Sample characteristics*

There were 53 patients in this study. Table 1 shows the characteristics of the study population, which had a median age of 51 years (range, 22–71 years). There were 15 patients (28%) who had stage II, 31 patients (59%) who had stage III, and 7 patients (13%) who had stage IV EOC. The histologic variants included the following: 19 serous (36%), 17 mucinous (32%), 9 endometrioid (17%), and 8 clear cell (15%). Following the primary debulking surgery, 32 patients (60.4%) had suboptimal debulking and only 21 patients (39.6%) had optimal debulking. The median blood loss was 1,000 (range, 100–8,000), the median amount of ascites was 400 (range, 10–18,000), and the median body mass index was 21 (range, 10.34–34.7).

*FASN and CA 125 serum levels*

Compared with the optimal debulking group, the suboptimal debulking group had significantly higher mean FASN serum level (0.36 ± 0.128 ng/mL vs 0.46 ± 0.144 ng/mL, p = 0.012); mean CA 125 serum level (964.22 ± 1722.5 U/mL vs. 264.98 ± 251.8 U/mL, p = 0.002); median serum FASN level [0.44 ng/mL (range, 0.21–0.77 ng/mL) vs. 0.34 ng/mL (range, 0.17–0.73 ng/mL, p = 0.01)]; and median serum CA 125 level [600 U/mL (range, 4.29–9934 U/mL) vs. 132.7 U/mL (range, 5.1–701 U/mL, p = 0.002)].

As shown in Table 3, the cutoff values were 0.375 for serum FASN level, with 68.8% sensitivity, 61.9% specificity, and 66% accuracy (p = 0.028); 222.50 for CA 125 level, with 75% sensitivity, 61.9% specificity, and 69.8% accuracy (p = 0.007); and 471.06 for the combination of FASN and CA 125, with 68.8% sensitivity, 71.4% specificity, and 69.8% accuracy (p = 0.004). As shown in Figures 1–3, the ROC analysis obtained AUC of 71.3% (95% CI 56.8%–85.8%, p = 0.009) for FASN; AUC of 75.3% (95% CI 62.5%–88.1%, p = 0.002) for CA 125; and AUC of 77.5% (95% CI 65%–89.9%, p = 0.001) for the combination of FASN and CA 125.

## Discussion

The result of debulking or the size of residual tumor after EOC surgery is a critical factor for a patient’s life expectancy (Holschneider, 2000). Optimal debulking has been associated with sensitivity to chemotherapy and survival rate (Bristow et al., 2002). In one study, immunohistochemical examination found no FASN expression in normal ovarian cells, but excessive expression of FASN was found in ovarian cancer, especially in the advanced stages (Cai et al., 2015). In this study, the mean serum level of FASN was higher with suboptimal debulking than with optimal debulking. Based on our results, patients who had FASN levels higher than the cutoff value of 0.375 were more likely to have >1 cm residual tumor after debulking (i.e., suboptimal), with 68.8% sensitivity, 61.9% specificity, 66% accuracy and AUC of 71.3% (95% CI 56.8% to 85.8%). The results of this study supported the theory on the association of increased FASN expression with advanced stage and the tendency to result in worse overall survival rates. In 2015, the research conducted by Cai et al found that 67 of 95 (70.5%) patients had high FASN expression in the tumor cell cytoplasm and tended to have worse overall survival rates (Memarzadeh et al., 2003; Cai et al., 2015).

In this study, the mean CA 125 level was significantly higher in the suboptimal debulking group than in the optimal debulking group. Moreover, patients who had CA 125 levels higher than the 222.50 cutoff value were 75.3% more likely to have suboptimal debulking surgery, with 75% sensitivity, 61.9% specificity, 69.8% accuracy, and AUC of 75.3% (95% CI 62.5% to 88.1%). The use of serum CA 125 level as a predictor of debulking surgery in advanced EOC remains controversial because of its low sensitivity and specificity.(Memarzadeh et al., 2003)

The new cutoff for the combination of FASN and CA 125 was 471.06, with 68.8% sensitivity, 71.4% specificity, 69.8% accuracy (p = 0.004), and AUC of 77.5% (95% CI 65.5%–89.9%, p = 0.001). The combined value of CA-125+FANS is obtained by multiplying the CA-125 numeric value and the FASN categorical value. At the serum FASN level value <0.375, the value is 1, while the serum level value> 0.375 is 2. Then the categorical value on the FASN is multiplied by the numerical value on CA-125. If the combined value <471.06, the patient will tend to undergo optimal debulking surgery; conversely, if> 471.06, the patient will tend to undergo suboptimal debulking surgery.

The combination of serum FASN and CA 125 levels may help predict who among patients with advanced EOC would be suboptimal or optimal debulking candidates. This combination was associated with suboptimal debulking surgery and had higher AUC and specificity, compared with those of FASN and CA 125 alone. Therefore, the combination of FASN and CA 125 can be used to predict the results of debulking surgery and help oncologists determine further therapy. The significant increase in FASN expression in patients with suboptimal surgery has been correlated with the existing theory on a more aggressive cancer phenotype in the presence of FASN expression and excessive activity, which can affect EOC progression and recurrence (Ameer et al., 2014),(Ueda et al., 2010a). Moreover, increased expression of FASN was shown to induce transition of the epithelium, thereby, causing cancer growth, increased number of metastatic ovarian cancer cells in the peritoneal cavity, and less success of tumor surgery (Jiang et al., 2014). High FASN expression was demonstrated in as many as 94.1% of patients with FIGO stage IV ovarian cancer but in only 1.25% of patients with stage I ovarian cancer (Ueda et al., 2010b; Cai et al., 2015).

Notably, all clinical and imaging methods should still be used to predict the result of debulking surgery. One limitation of our study was the small sample size; therefore, the power to detect significant differences between suboptimal and optimal debulking may have been limited. Nevertheless, this research may help oncologists better predict the results of debulking surgery in patients with EOC.

In conclusion, high preoperative value of the combination of FASN and CA 125 was correlated with suboptimal debulking surgery.
